# High-performance phononic crystal sensing structure for acetone solution concentration sensing

**DOI:** 10.1038/s41598-023-34226-4

**Published:** 2023-04-29

**Authors:** Tian-Yin Fang, Xiao-Wei Sun, Xiao-Dong Wen, Yun-Xia Li, Xi-Xuan Liu, Ting Song, Yu-Zhe Song, Zi-Jiang Liu

**Affiliations:** 1 School of Mathematics and Physics, Lanzhou Jiaotong University, Lanzhou, 730070 China; 2 Institute of Sensor Technology, Gansu Academy of Sciences, Lanzhou, 730070 China

**Keywords:** Acoustics, Mathematics and computing

## Abstract

A two-dimensional phononic crystal sensor model with high-quality factor and excellent sensitivity for sensing acetone solutions and operating at 25–45 kHz is proposed. The model for filling solution cavities is based on reference designs of quasi-crystal and gradient cavity structures. The transmission spectrum of sensor is simulated by the finite element method. High-quality factor of 45,793.06 and sensitivity of 80,166.67 Hz are obtained for the acetone concentration with 1–9.1%, and quality factor of 61,438.09 and sensitivity of 24,400.00 Hz are obtained for the acetone concentration range of 10–100%, which indicated the sensor could still achieve high sensitivity and quality factor at operating frequencies from 25 to 45 kHz. To verify the application of the sensor to sensing other solutions, the sensitivity for sound velocity and density is calculated as 24.61 m^-1^ and 0.7764 m^3^/(kg × s), respectively. It indicates the sensor is sensitive to acoustic impedance changes of the solution and equally suitable for sensing other solutions. The simulation results reveal the phononic crystal sensor possessed high-performance in composition capture in pharmaceutical production and petrochemical industry, which can provide theoretical reference for the design of new biochemical sensors for reliable detection of solution concentration.

## Introduction

Phononic crystals (PnCs) is periodic structures that are engineered to manipulate the propagation of acoustic or elastic waves^1^. PnCs with phononic band gap properties prevent the propagation of elastic waves in a specific frequency range. Based on this property, many researchers have achieved the manipulation of acoustic waves by PnCs^2–9^. For example, environmental noise control, acoustic clock and making acoustic devices with switches, negative refraction, directional radiation, sensors and other functions^2–7^. Among them, PnC sensor is a new device that can sense solutions without using any electronic components in the detection process. Compared with other sensors, such as plasma sensors and photonic crystal sensors, the PnC sensors have higher sensitivity for the detection of solution concentration owing to the variation of sound velocity and density of the solution with concentration is more obvious^7^. The PnC sensor is mainly based on the band gap properties through the Anderson localization effect generated by defect states. Wu et al. investigated defect states in mercury based PnCs by numerical simulations. And they introduce defects in the perfect periodic structure to create Anderson localization effects^10^. Thus, PnC sensor can distinguish different solutions concentrations by the different resonance frequencies appearing at the measuring solution (defects). Zubtsov et al. experimentally prepared a PnC sensor using a water/propanol mixture as the measured solution for the first time, verifying the feasibility of the PnC sensor^11^.

PnC sensor is mainly divided into one-dimensional (1D) laminar sensor and two-dimensional (2D) plate sensor. For 1D layered PnC sensor, the measured target, such as the tested solution, functional material, as a defect was added to the periodically arranged PnC, and the acoustic properties of the measured target were evaluated by calculating and analyzing the transmission spectrum. In addition, functional materials can be added to the sensor as defect to increase the sensing physical quantities of the sensor, such as magnetic field strength and pressure. Mehaneg studied the relationship between the thickness, temperature and pressure of the biodiesel layer and the shift of the resonance peak by the transfer matrix method^12^. He and collaborator measured the concentration of the acetone solution with irregularly changing sound velocity at different concentrations^13^. The team designed the sensor mainly based on the transfer matrix method using 1D laminar structure to sense solutions with different characteristics. The simulation calculations results showed that the PnC sensor could be used to sense different characteristics solutions. Nagaty et al. introduced a piezomagnetic material layer as a defect in 1D layered PnC^14^. It extends the detection range of PnC sensors to include magnetic field measurements and pressure detection. Because the performance of the sensors just can be optimized by changing the thickness of layer and replacing materials, the 1D layer structure is hard used in practice. In contrast, 2D PnC plate sensor can be more flexibly optimized for acoustic sensing performance through changes in the defect arrangement structure, such as the introduction of gradient cavities and quasi-crystal arrangements. For 2D plate PnC sensor, Hamed designed a 2D plate PnC sensor with a gradient cavity at the measured solution and measured the hydrogen peroxide solution concentration^15^. The introduction of the gradient cavity makes the acoustic energy converge to the measured solution more effectively. Therefore, it significantly improved the quality factor and sensitivity of the sensor. Ayna et al. added a piezoelectric transducer as the operating excitation of the PnC sensor and arranged the single cells in a quasi-crystal manner during the simulation, resulting in a transmission spectrum quality factor of 40,000^16^. However, there are still problems, such as the high working frequency of the sensor operating excitation, the good performance requirements of the operating excitation, the need for high frequency acoustic waves to drive the sensor to maintain a high-quality factor and high sensitivity. These problems need to be further addressed by researchers.

Acetone as chemical reagent is massive produced and widely used in laboratories and industry, where it is used as a solvent and to produce methyl methacrylate^17,18^. Acetone has a very wide range of industrial uses in the production of plastics. Acetone is also the first choice for laboratory cleaning. Besides, the number of seizures in infants and children with severe epilepsy can be reduced by increasing the concentration of acetone that is produced in the body through a ketogenic diet. In addition, among the hundreds of volatile organic compounds breathed out by humans, the acetone concentration in exhaled breath is closely related to the concentration of glucose and acetone in the blood, so acetone is also a biomarker for 1 type diabetes^19–21^. However, the detection of the concentration of acetone solution still requires titration and gas chromatography. So, there is a great need to detect the concentration of acetone solution by a low-cost and highly sensitive detection method in life.

Based on these, we designed a novel PnC sensor for sensing acetone solution. Our research is based on finite elements method (FEM). A two-dimensional PnC resonator is constructed by introducing a gradient cavity and a quasi-crystal structure to sense acetone. Sensor performance parameters, such as sensitivity, the quality factor and figure of merit, are calculated analytically for all concentrations. Finally, it is compared with other PnC sensors performance parameters.

## Model and method

In this work, mercury and water are chosen as the substrate and scatterer, respectively. The mechanical parameters of the materials, such as density (*ρ*), velocity of sound (*c*) and bulk modulus of elasticity (*B*), are shown in Table 1.

**Table 1:** Mechanical parameters of mercury and water for the model's substrate and scatterer materials.

Material	*ρ* (kg/m^3^)	*c* (m/s)	*B* (GPa)
Mercury	13,500	1450	28.5
Water	1000	1490	2.15

**Figure 1:**
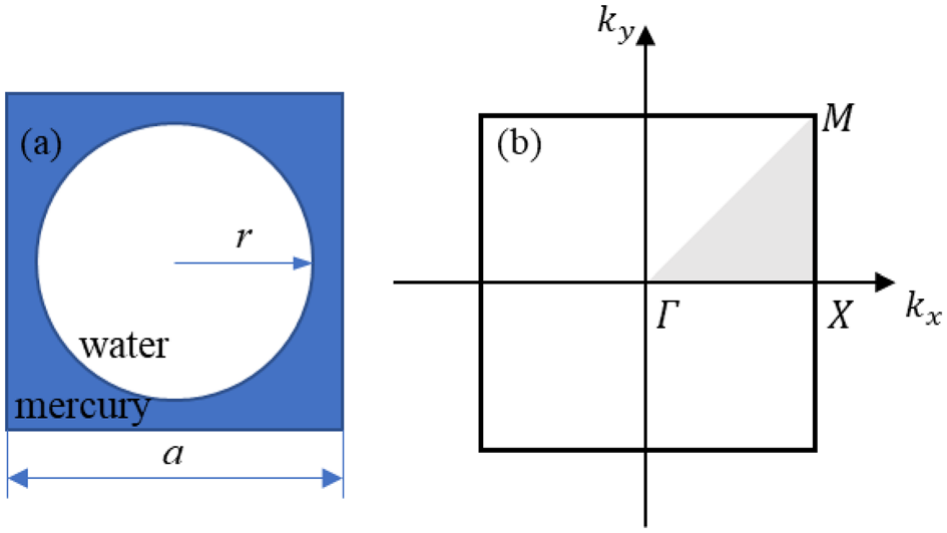
PnC unit cell structure, where (**a**) is PnC unit cell and (**b**) is irreducible brillouin zones and high symmetry edges.

According to the Bloch-Floquet law, the wave vectors (***k***_x_, ***k***_y_) are analyzed on the symmetry edge (ΓΧΜ) of the integrable Brillouin zone and Eigenvalues are calculated. The single cell lattice constant (a) is 20 mm, and the scatterer radius (r) is 6 mm. The ideal single cell structure with a scatterer (water) fill rate of 0.2827 is showed in Fig. 1. When calculating the energy band diagram, the periodic boundary conditions are placed on all boundaries of the structure^22,23^.

**Figure 2:**
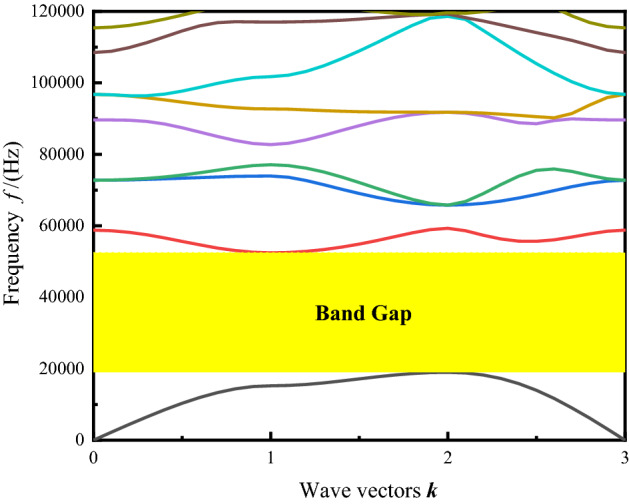
The band gap of PnC unit cell for the designing sensor by FEM.

Band gap is a fundamental property of PnCs, which are ideal perfectly periodically arranged structures. It can be designed and optimized by replacing materials and changing the structure. The band gap width obtained from the single cell is 33 kHz, and the band gap frequency belongs to the frequency range of ultrasonic, as shown in Fig. 2.

**Figure 3:**
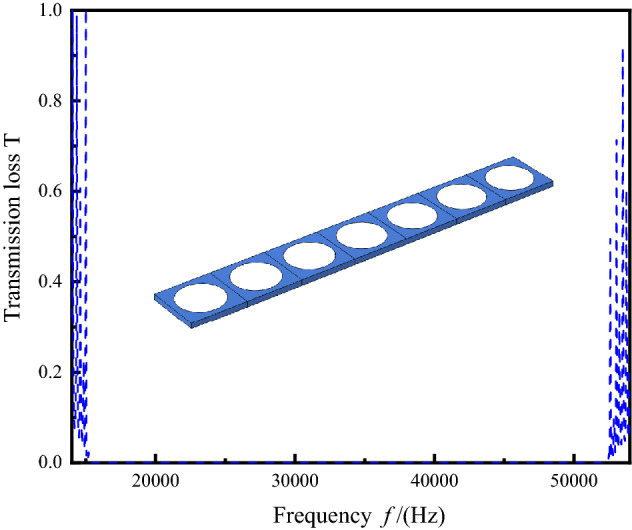
Transmission loss of a 1 × 7 perfect supercell.

A PnC single-cell band gap is shown in Fig. 2, which is caused by a large difference between the material elastic constants^9,24^. The phononic band diagram depicts a band gap up to 33 kHz. To verify the calculation results of the single cell band gap, the transmission loss of 1 × 7 perfect supercell is also calculated and plotted, as shown in Fig. 3. Figures 2 and 3 show the same band gap with a band gap ranging from 19 to 52 kHz.

**Figure 4:**
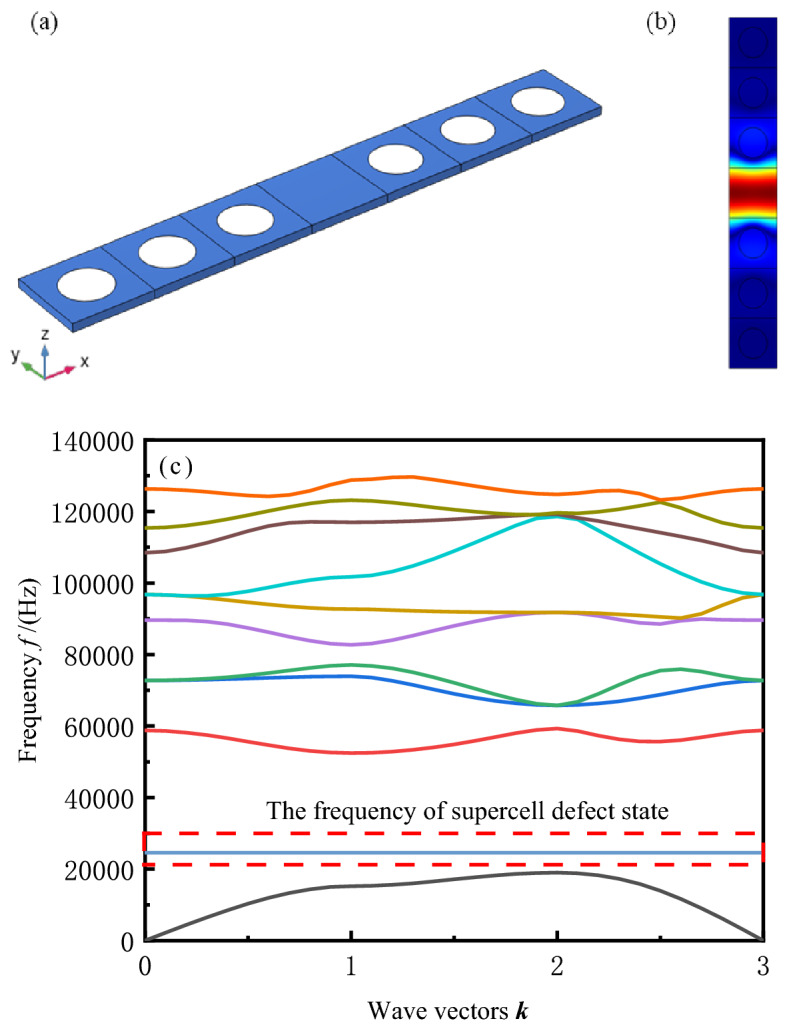
PnC supercell defect state where (**a**) is 1 × 7 supercell structure diagram with one defect, (**b**) is Anderson localization effect of defect states at 24,560 Hz, and (**c**) is supercell defect band.

To propagate sound waves in the measured liquid, a waveguide channel needs to be constructed in a regular and periodic PnC structure. In this work, waveguiding is achieved by removing a row of scatterers in the entire PnC plate structure to form a line defect. In fact, the work principle of PnC sensors is based on the presence of line defects in the structure and the generation of a series of defect bands in the bandgap region. And then the transmission of acoustic energy from the Anderson localization effect localization through the waveguide generated by the line defects to resonance and exit at the tested solution. Therefore, we need to filter the acoustic waves with frequencies associated with these defect bands so that they can propagate in the highly confined waveguide channel. A sound wave with a certain characteristic frequency collides with the sensing area designed as a resonator and is emitted in the path of the sensor. The liquid concentration is sensed by analyzing the frequency of the outgoing sound wave. The resonator was consisted with a large hole as a cavity with several scatterers surrounding it, and the cavity containing the sensed target solution. Depending on the acoustic properties of the target solution (e.g., density and velocity of sound), the resonator will have a specific resonant frequency. When a plane wave of the same frequency as the resonant cavity is input, the plane wave will be enhanced in the resonant cavity and emitted through the PnC waveguide channel. The resonant frequency of the resonant cavity will be affected by the acoustic properties of the added measured solution. Therefore, the PnC sensor can determine the solution concentration by judging the acoustic properties of the solution according to the output acoustic frequency. To verify that whether a defect state can be generated in the band gap, a 1 × 7 supercell with defects is constructed and calculated. A defect energy band with a frequency of 24,560 Hz is found and appear in the band gap frequency range. The acoustic energy converges at the defects, and the supercell model, energy localization effect and defect state energy band are shown in Fig. 4. Figure 4 shows that only the acoustic energy at this frequency can propagate at the defect with converging at the defect.

**Figure 5:**
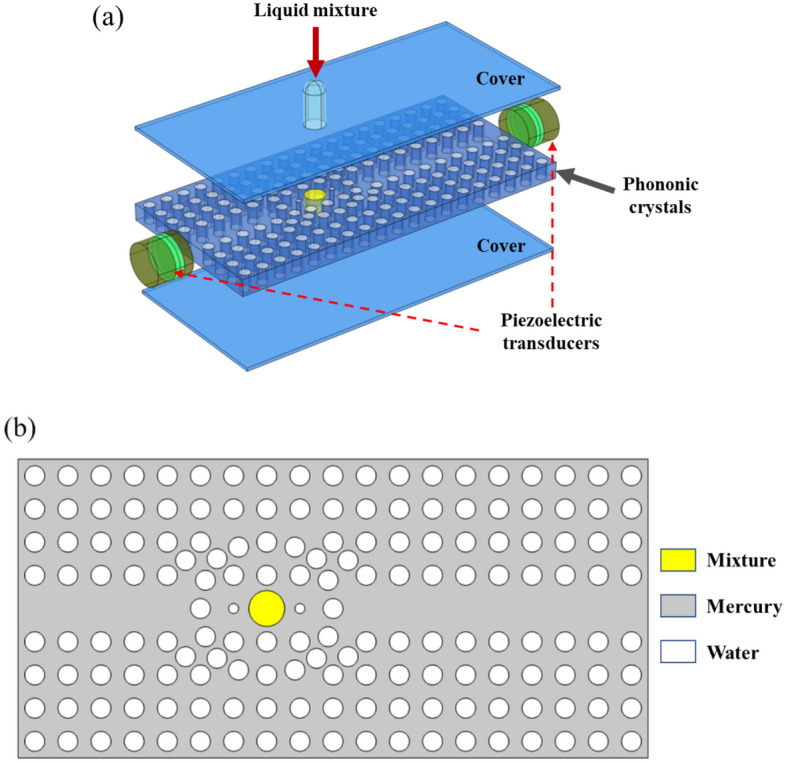
The structures and materials of PnC sensor where (**a**) is a device for PnC sensor to sense solution and (**b**) is two-dimensional plate-like PnC sensor structure (These figures are made by COMSOL Multiphysics 6.1^25^).

The designed PnC sensor is consisted with an array of single cells, the substrate is constructed by mercury and the scatterer is filled with water. Subsequently, some scatterers are removed in the middle of the sensor and a resonant cavity is formed around the yellow part in the middle to produce a resonance phenomenon with the measured solution, as shown in Fig. 5. Only acoustic waves of specific frequencies are allowed to pass through the waveguide channel portion of the PnC sensor. According to the single-cell band gap, the working frequency of the sensor is in the ultrasonic frequency range of 19 kHz to 52 kHz. To obtain plane waves in frequency range within the waveguide channel, a longitudinal piezoelectric transducer is selected as the excitation in this work. To keep the waveform unchanged when emitting and facilitate measurement, the emitting channel of the sound wave is extended. The first-order resonant frequency of the longitudinal piezoelectric transducer is negatively correlated with its longitudinal length. And when the resonant frequency of the transducer increases, the difficulty in fabrication process of driver circuit increases^26^. Therefore, the operating frequency of the PnC sensor is selected from 19 to 52 kHz for practicality. To separate the water from the mercury, an ultra-thin solid rubber shell is used to maintain the stability of the liquid–liquid system. Theoretical and experimental studies have shown that the ultrathin solid rubber shell structure has almost no effect on device operation and performance^27,28^.

With the presence of incident acoustic pressure field in the sensor structure, the acoustic pressure equation was considered in the whole structure as^15,22,23^

1 $$\nabla \cdot \left( {\frac{1}{{\rho c\left( {\nabla p_{t} - q_{d} } \right)}}} \right) - \frac{{k_{eq}^{2} p_{t} }}{\rho c} = Q_{m} ,$$

2 $$k_{eq}^{2} = \left( {\frac{\omega }{c}} \right)^{2} - k_{z}^{2} ,$$

where *ρ* is the density, *c* is the velocity of sound, *k*_*eq*_ is the equivalent wave number, *k*_*z*_ is the out-of-plane wave number, *Q*_*m*_ is the monopole source, *q*_*d*_ is the dipole source, *p*_*t*_ is the total sound pressure, and *ω* is the angular frequency, respectively.

The equations related to the acoustic transmission of the mercury substrate and water as a scatterer are expressed as follows^13,20,21^

3 $$- \nabla p\left( {r,t} \right) = \rho^{^{\prime}} \cdot \frac{{\partial v\left( {r,t} \right)}}{\partial t},$$

4 $$\frac{{\partial p\left( {r,t} \right)}}{\partial t} = - B\left( r \right)\nabla \cdot v\left( {r,t} \right),$$

where *ρ'* is the density of the medium after the generation of acoustic perturbation, *v* is the medium mass velocity, and *B* is the bulk modulus of elasticity, respectively.

## Results and discussion

### Physical properties of acetone

**Figure 6:**
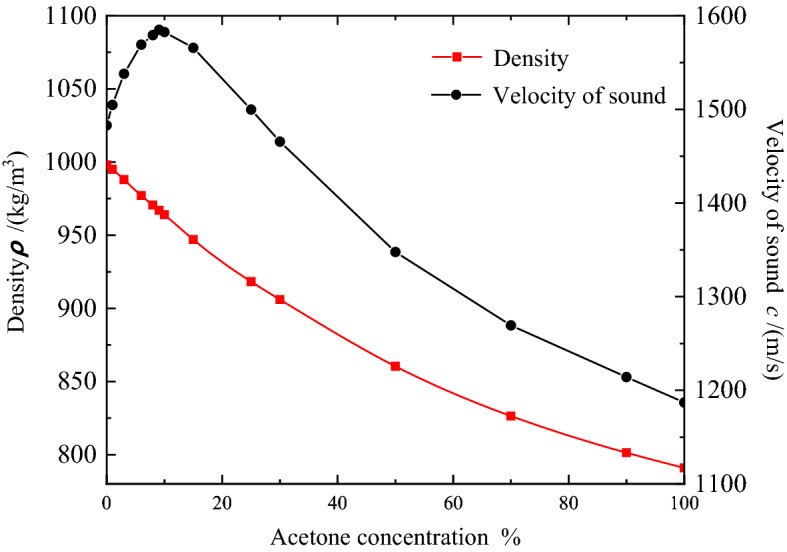
Velocity of sound and density of the 0–100% acetone concentration in water (vinegar mixture).

The designed PnC sensor is mainly used to sense acetone solution concentration. Based on experimental results of reference 13, the exact acoustic parameter values of acetone are plotted. Figure 6 shows the variation curves of physical properties, such as sound velocity and mass density of acetone. The acoustic properties of acetone change with concentration. With the increase of acetone concentration (1—9.1%), the sound velocity gradually increases and reaches a maximum at the concentration of 9.1%. When the acetone concentration is further increased, the sound velocity decreased with the increasing of acetone concentration. When the concentration is more than 9.1%, the solubility of acetone in water started to change. Therefore, the relationship between the speed of sound and acetone concentration showed an opposite change. With the concentration of 9.1% or more, acetone is completely dissolved in water, and water is saturated with acetone. Hydrogen bonds are no longer formed between water/acetone molecules, and the sound velocity change was affected by the intermolecular forces change^29,30^. Therefore, using pure water as a reference, we divide the into two parts (low concentration (1–9.1%) and high concentration (10–100%)) to calculate the sensor performance parameters.

### Sensor performance analysis

There are many parameters to evaluate the sensing performance of the sensor, the most important of which is the sensitivity (*S*_*n*%_). The formula of sensitivity about concentration change is calculated as follows^31^,

5 $$S_{n\% } = \frac{\Delta f}{{\Delta n}},$$

Where $\Delta f$ is the absolute value of the difference between the resonant peak frequency generated by each acetone concentration and the resonant frequency of the zero-concentration solution (water), $\Delta f = \left| {f_{n\% } - f_{0\% } } \right|$, $\Delta n$ is concentration difference. The resonant frequency of pure water is used as the reference value.

When analyzing sensor performance, the quality factor (*Q*_*f*_) is another important parameter and can be calculated by the following relationship,

6 $$Q_{f} = \frac{{f_{r} }}{{\Delta f_{{{\raise0.7ex\hbox{$1$} \!\mathord{\left/ {\vphantom {1 2}}\right.\kern-0pt} \!\lower0.7ex\hbox{$2$}}}} }}.$$

*f*_*r*_ indicates the resonates frequency produced by acetone solution in the cavity, and $$\Delta f_{{{\raise0.7ex\hbox{$1$} \!\mathord{\left/ {\vphantom {1 2}}\right.\kern-0pt} \!\lower0.7ex\hbox{$2$}}}}$$ is the full widths at half maximum at the resonant frequency. Beside the sensitivity and the quality factor, the figure of merit (FOM) is also an important parameter and can be obtained from the following relationship,

7 $${\text{FOM}} = \frac{{S_{n\% } }}{{\Delta f_{{{\raise0.7ex\hbox{$1$} \!\mathord{\left/ {\vphantom {1 2}}\right.\kern-0pt} \!\lower0.7ex\hbox{$2$}}}} }}.$$

Three parameters mentioned above indicate the sensing performance of the PnC sensor. We calculate the transmission spectrum of concentration 0–100% acetone solution by finite element method. And the performance of the sensor is analyzed in relation to the individual resonance peaks of the transmission spectrum.

**Figure 7:**
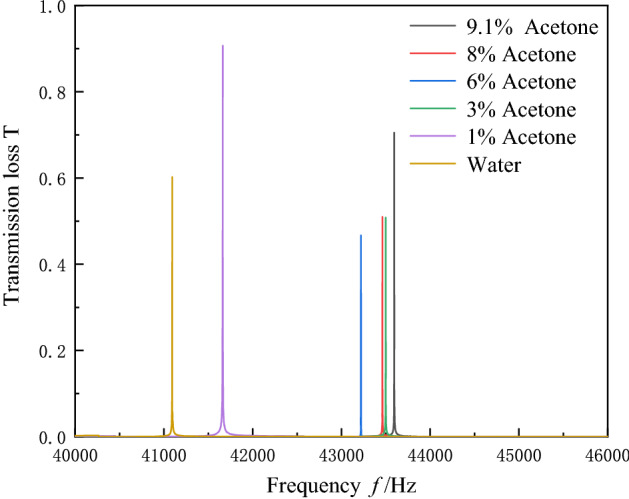
Acoustic transmission spectrum of 0—9.1% acetone solution sensed by PnC sensor.

According to the investigation of acetone solutions in 2.2, it is found that the changing trends of sound velocity and density are different in the concentration ranges of 1–9.1% and 10–100%. Therefore, the analysis of the sensor performance in this work is divided into two parts: 1–9.1% and 10–100% concentration range. In order to make the simulation calculation closer to the actual experiment, we consider the viscosity of both water and mercury. We determine the viscosity for both materials with the values of 0.0015 and 0.00089 (Pa × s), respectively. Firstly, the transmission spectra are calculated for the concentration range of 1–9.1%, as shown in Fig. 7. The resonance peaks found in the transmission spectrum with various concentration solution can be clearly distinguished from each other. The resonance peaks of the 3% and 8% concentrations are very close to each other. The resonance frequency does not increase with as concentration growth. Within the concentration range of 1–9.1%, the density decreases, as well as the speed of sound increases along with the increasing in concentration. However, the change in acoustic impedance cannot be seen directly by the change in speed of sound or density. By the calculating, the acoustic impedance changes abruptly when the solution concentration is 6%. According to Fig. 6, the density of acetone solution with a concentration of 3% is 987.9677 kg/m^3^ and the speed of sound is 1,538.0323 m/s. The density of acetone solution with 8% concentration is 970.5826 kg/m^3^ and the speed of sound is 1,579.3721 m/s. The acoustic impedances of the 3% and 8% solutions are similar to each other, so the resonance peaks of the 3% and 8% solutions are very close to each other. The performance parameters of the sensor are calculated based on the transmission spectrum, and the results are shown in Table 2. The quality factor of the resonance peaks at 3% solution concentration was reaches at a peak of 45,793.06 and the corresponding sensitivity is 80,166.67 Hz. The quality factors of the resonance peaks at other concentration solutions were above 19,000 at the corresponding operating frequencies. This demonstrates that the PnC sensor is capable of excellent acoustic energy convergence at lower operating frequencies while maintaining high sensitivity.

**Table 2:** Performance parameters of PnC sensor when sensing 0—9.1% acetone concentration.

*n* (%)	Resonant Frequency (Hz)	*Q*_*f*_	*S*_*n*%_ (Hz)	FOM
0	41,094	24,315.98	–	–
1	41,666	19,388.55	57,200.00	26,617.03
3	43,499	43,525.12	80,166.67	80,214.80
6	43,220	37,589.15	35,433.33	30,816.96
8	43,462	45,667.75	29,600.00	31,102.24
9.1	43,595	45,793.06	27,483.52	28,869.24

**Figure 8:**
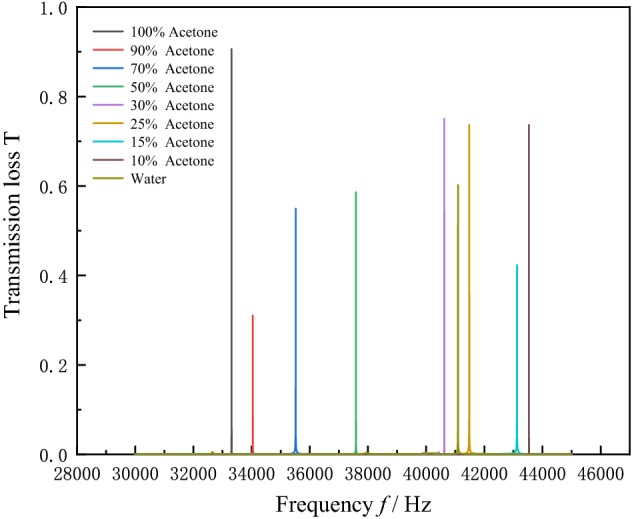
Acoustic transmission spectrum of 10–100% acetone solution sensed by PnC sensor.

**Figure 9:**
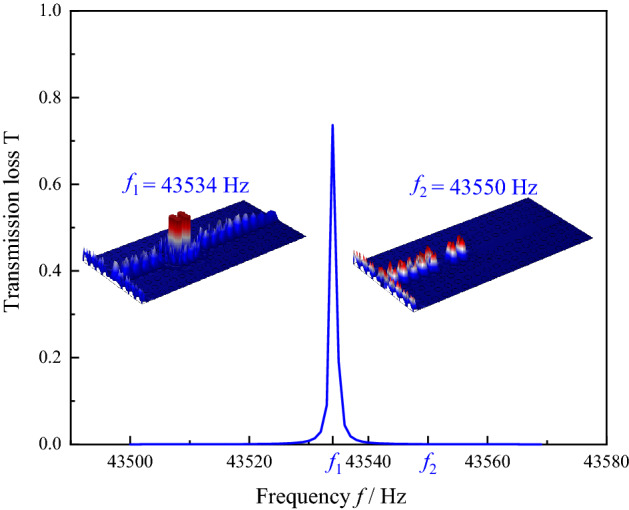
Sound field distribution in the sensor when sensing 10% solution concentration (These figures are made by COMSOL Multiphysics 6.1^25^).

The transmission spectrum of sensor is calculated for the 10–100% range of solutions concentration, as shown in Fig. 8. The sensor performance parameters corresponding to the resonance peaks for each concentration are calculated, as shown in Table 3. The resonance peaks at 25% and 30% are found to be very close to those of water by transmission spectroscopy, resulting in a low sensitivity for sensing solutions concentration at 25% and 30%. Nevertheless, the characteristic frequencies of resonance peak of the transmission spectrum can be used to distinguish the various solutions concentrations. The waveguide sound field distribution is calculated as a solution concentration of 10%, as shown in Fig. 9. The sound field distribution of defective state resonant frequency *f*_1_ and non-defective state resonant frequency *f*_2_ can be seen in Fig. 9. The acoustic energy converges at the measured solution and is emitted as a plane wave at *f*_1_ frequency. While the acoustic wave converges at the measured solution but cannot be emitted due to the limitation of PnC wall at *f*_2_ frequency. Only when the frequency of the input sound wave is consistent with the resonance frequency corresponding to the solution of this concentration, the sound wave of this frequency can be emitted.

**Table 3:** Performance parameters of PnC sensor when sensing 10–100% acetone concentration.

*n* (%)	Resonant Frequency (Hz)	*Q*_*f*_		*S*_*n*%_ (Hz)	FOM
0	41,094	24,315.98		–	–
10	43,534	43,373.52		24,400.00	24,310.05
15	43,121	18,043.01		13,513.33	5654.35
25	41,483	61,438.09		1566.00	2304.50
30	40,624	26,570.74		1566.67	1024.70
50	37,587	33,422.55		7014.00	6236.88
70	35,512	16,143.92		7970.00	3622.89
90	34,040	35,835.35		7837.77	8251.16
100	33,322	17,071.57		7772.00	3981.76

**Figure 10:**
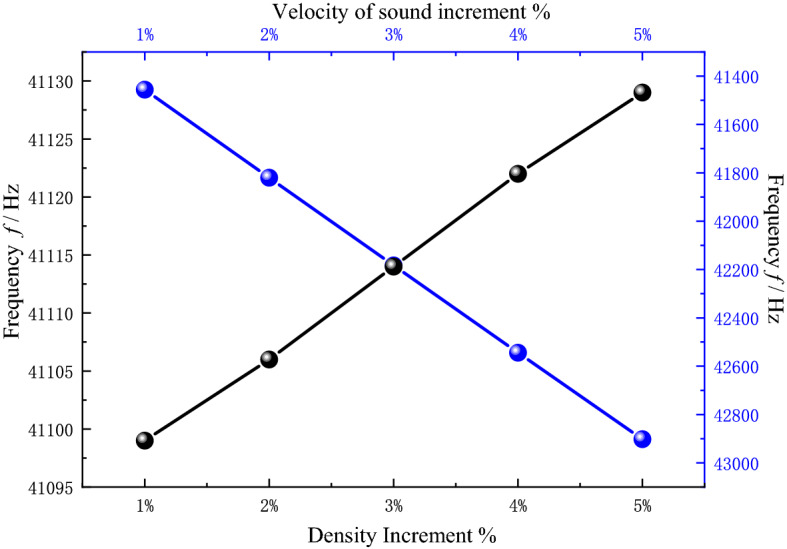
The relationship of each resonance frequency generated by increasing the sound speed and density by 1–5% based on water.

To verify the applicability of PnC sensor to other solutions, the sensitivity of the sensor to sound velocity and density is also calculated. By using water as the base, the resonant frequencies are calculated with the increasing of speed and density of sound by 1–5% respectively, and the results are shown in Fig. 10. The resonant frequency increases with the increasing in the velocity of sound and density. The shift of frequency is 364 Hz for a 1% increasing in velocity of sound and 7.49 Hz for a 1% increasing in density. As shown in Fig. 10, the sensor has a sensitivity of 24.58 m^-1^ for sound velocity and 0.7764 m^3^/(kg × s) for density. The sound wave of a specific frequency can only be emitted when the impedance of the solution to be tested matches that of the substrate boundary. The impedance of the boundary is proportional to the product of the sound velocity and density of the solution to be measured and is proportional to the product of the surface density and frequency of the substrate at the boundary. So, when the speed of sound or density increases, the frequency of the transmitted sound wave also increases. The results show that the PnC sensor can discriminate at a 1% change in sound velocity and density, fully demonstrating the sensor's ability to sense other solutions and to discriminate solution concentrations.

Finally, we compare the sensitivity of our sensor with previous 1D and 2D PnC sensors in Table 4. As shown in the table, the emphasis is on comparing the 1D PnC of the literature 11 which also senses the acetone solution. We can see that the highest sensitivity of the acetone concentration sensor designed in literature 11 is 2.04 × 10^7^ Hz, and the range of operating frequency is 1.63–1.70 × 10^10^ Hz. Compared with the sensor designed in this paper, the 1D PnC sensor has higher sensitivity, but this is based on its ultra-high driving frequency. To approximate the effect of drive frequency on sensitivity, we can compare the ratio of sensitivity to drive frequency to determine which sensor is more sensitive. Through the comparison of this ratio, it is found that the sensor designed in this paper is more sensitive. While ensuring that the sensor is more sensitive, the driving frequency is reduced to a lower range of 33,322–43,595 Hz. In this paper, the performance optimization of the PnC sensor for the concentration of acetone solution is realized by combining the gradient cavity and the quasi-crystal structure.

**Table 4:** Comparison between the sensitivity of our design with previous published works.

Structure	Sensing material	Sensitivity	Sensor operating frequency
Acetone/water 1D phononic crystal sensor^13^	Acetone	High concentration 2.04 × 10^7^ Hz	1.63–1.70 × 10^10^ Hz
1D Locally resonant porous phononic crystal^32^	CdBr_2_ concentrations	47.25 Hz/ppm	1.70190237–1.7015982 × 10^8^ Hz
1D Phononic crystal from lead and epoxy multilayer with defect layer from gasoline components^33^	n-heptane	4.36301 × 10^11^ Hz/(Pa × s)	9.65 × 10^8^ Hz
	Cyclo-pentane	2.48596 × 10^11^ Hz/(Pa × s)	9.55 × 10^8^ Hz
	Isooctane	4.16009 × 10^10^ Hz/(Pa × s)	9.34 × 10^8^ Hz
	Ethanol	2.0096 × 10^10^ Hz/(Pa × s)	9.42 × 10^8^ Hz
1D phononic crystal from Aluminum and epoxy multilayer with defect layer from biodiesel fuel^34^	Methyl soy ester	34.14 m^−1^	175,176.8–183,090.2 Hz
	Oxidized soy ester	35.39 m^−1^	175,132.9–183,009.3 Hz
	Ethyl soy ester	36.02 m^−1^	174,826.9–182,788.1 Hz
	Certified D-2	43.88 m^−1^	171,954.4–180,570.9 Hz
	Methyl laurate	50.37 m^−1^	169,825.9–179,617.4 Hz
2D Phononic crystal locally-resonant cavity of quasi-crystal structure^16^	Vinegar	5300 Hz	191,872–192,363 Hz
2D phononic crystals combining gradient cavities and quasi-crystal structures (The present work)	Acetone	High concentration 80,166.67 Hz, 24.61 m^−1^	33,322–43,595 Hz

## Conclusions

In this work, a 2D PnC sensor for sensing acetone/water solution is designed with reference to quasi-crystal structure and gradient cavity structure, and the transmission spectrum of the sensor is simulated and calculated based on the finite element method. Different solutions concentrations have different acoustic impedances, and the incident acoustic waves will resonate at different frequencies for the measured solutions in the resonant cavity with different acoustic impedances, which will allow the sensor to accurately sense small changes in acetone concentration. A quality factor of 45,793.06 is achieved and a sensitivity of up to 80,166.67 Hz is maintained for the acetone solution in the concentration range of 1–9.1%, and a quality factor of 61,438.09 and a sensitivity of 24,400.00 Hz are obtained for the concentration range of 10–100%. The sensor differentiates the concentration of acetone solutions in the relative low ultrasound frequency range of 25–45 kHz and achieves both a high-quality factor and high sensitivity. In addition, the sensitivity of the sensor to sound velocity and density is also calculated as 24.61 m^−1^ and 0.7764 m^3^/(kg × s), which well illustrates that the sensor is also well suited for sensing other solutions as well as solution concentrations. The sensor designed in this paper does not use electronic components in the detection process and is easy to manufacture and apply in fluid systems. Compared with other sensors, the sensor designed in this paper can realize high-performance solution concentration sensing at a lower frequency. The results indicate that the PnC sensor could have promising applications in various industries, such as pharmaceutical production, petrochemicals, and cosmetics, for capturing ingredients. Additionally, these findings can offer theoretical guidance for the development of new biochemical sensors.

## Data Availability

The data that support the findings of this study are available from the corresponding author upon reasonable request.

## References

[1] Khelif, A. & Adibi, A. Phononic crystals. *Springer* **10**, 978-971 (2015).

[2] Huang J, Liu Y, Li Y (2019). Trees as large-scale natural phononic crystals: Simulation and experimental verification. Int. Soil. Water. Conse..

[3] Zheng L-Y (2014). Acoustic cloaking by a near-zero-index phononic crystal. Appl. Phys. Lett..

[4] Motaei F, Bahrami A (2020). Nonlinear elastic switch based on solid–solid phononic crystals. J. Mater. Sci..

[5] Sukhovich A, Jing L, Page JH (2008). Negative refraction and focusing of ultrasound in two-dimensional phononic crystals. Phys. Rev. B.

[6] Cheng C-W, Chen J (2016). Femtosecond laser sintering of copper nanoparticles. Appl. Phys. A.

[7] Oseev A, Zubtsov M, Lucklum R (2013). Gasoline properties determination with phononic crystal cavity sensor. Sensor. Actuat. B-Chem..

[8] Li XF, Ni X, Feng L, Lu MH, He C, Chen YF (2011). Tunable unidirectional sound propagation through a sonic-crystal-based acoustic diode. Phys. Rev. lett..

[9] Moradi P, Bahrami A (2019). Three channel GHz-ranged demultiplexer in solid-solid phononic crystals. Chinese J. Phys..

[10] Wu F, Hou Z, Liu Z, Liu Y (2001). Point defect states in two-dimensional phononic crystals. Phys. Lett. A.

[11] Zubtsov M (2012). 2D phononic crystal sensor with normal incidence of sound. Sensor. Actuat. A: Phys..

[12] Mehaney A (2019). Biodiesel physical properties detection using one-dimensional phononic crystal sensor. Acoust. Phys..

[13] Mehaney A, Ahmed II (2021). Acetone sensor based 1D defective phononic crystal as a highly sensitive biosensor application. Opt. Quant. Electron..

[14] Nagaty A, Mehaney A, Aly AH (2018). Acoustic wave sensor based on piezomagnetic phononic crystal. J. Supercond. Nov. Magn..

[15] Gharibi H, Mehaney A (2021). Two-dimensional phononic crystal sensor for volumetric detection of hydrogen peroxide (H2O2) in liquids. Physica E.

[16] Khaligh A, Bahrami A, Ghavifekr HB (2021). Phononic crystal locally-resonant cavity for detecting vinegar acidity. J. Mol. Liq..

[17] Andrigo P (1992). Phenol-acetone process: cumene oxidation kinetics and industrial plant simulation. Chem. Eng. Sci..

[18] Georgiev G, Dakova I, Valova N (1994). Radical methyl methacrylate—Methacrylic acid copolymerization in isopropyl alcohol, acetone, and their mixtures. Application of the copolymer products for microencapsulation of ampicylline trihydrate. Colloid Polym. Sci..

[19] Kim SJ (2017). Exceptional high-performance of Pt-based bimetallic catalysts for exclusive detection of exhaled biomarkers. Adv. Mater..

[20] Storer M (2011). Measurement of breath acetone concentrations by selected ion flow tube mass spectrometry in type 2 diabetes. J. Breath Res..

[21] Zhang X (2020). Highly sensitive and selective acetone sensor based on three-dimensional ordered WO3/Au nanocomposite with enhanced performance. Sensor. Actuat. B-Chem..

[22] Gharibi H, Bahrami A (2020). Phononic crystals for sensing FAMEs with demultiplexed frequencies. J. Mol. Liq..

[23] Gharibi H, Khaligh A, Bahrami A, Ghavifekr HB (2019). A very high sensitive interferometric phononic crystal liquid sensor. J. Mol. Liq..

[24] Moradi P, Bahrami A (2018). Design of an optomechanical filter based on solid/solid phoxonic crystals. J. Appl. Phys..

[25] COMSOL Multiphysics® v. 6.1. cn.comsol.com. COMSOL AB, Stockholm, Sweden.

[26] Lin S (2004). Principle and design of ultrasonic transducer. Science Press.

[27] Pennec Y, Vasseur JO, Djafari-Rouhani B, Dobrzyński L, Deymier PA (2010). Two-dimensional phononic crystals: Examples and applications. Surf. Sci. Rep..

[28] Vasseur JO (1998). Experimental evidence for the existence of absolute acoustic band gaps in two-dimensional periodic composite media. J. Phys- Condens. Mat..

[29] Buhvestov U, Rived F, Ràfols C, Bosch E, Rosés M (1998). Solute–solvent and solvent–solvent interactions in binary solvent mixtures. Part 7. Comparison of the enhancement of the water structure in alcohol–water mixtures measured by solvatochromic indicators. J. Phys. Org. Chem..

[30] Sehgal CM, Porter BR, Greenleaf JF (1986). Ultrasonic nonlinear parameters and sound speed of alcohol–water mixtures. J. Acoust. Soc. Am..

[31] Ahmed AM, Mehaney A (2019). Ultra-high sensitive 1D porous silicon photonic crystal sensor based on the coupling of Tamm/Fano resonances in the mid-infrared region. Sci. Rep..

[32] Alrowaili ZA (2023). Locally resonant porous phononic crystal sensor for heavy metals detection: A new approach of highly sensitive liquid sensors. J. Mol. Liq..

[33] Mehaney A, Hassan MS, Elsayed HA (2021). Fuel phononic crystal sensor for the determination and discrimination of gasoline components. Plasmonics.

[34] Mehaney A (2020). Temperature influences on the performance of biodiesel phononic crystal sensor. Mater. Res. Express..

